# Developmental plasticity of bacterial colonies and consortia in germ-free and gnotobiotic settings

**DOI:** 10.1186/1471-2180-12-178

**Published:** 2012-08-15

**Authors:** Irena Pátková, Jaroslav J Čepl, Tomáš Rieger, Anna Blahůšková, Zdeněk Neubauer, Anton Markoš

**Affiliations:** 1 Department of philosophy and history of Science, Faculty of Science, Charles University in Prague, Viničná 7, Praha 2, Czechia, 12844

**Keywords:** Ontogeny of bacteria, Germ-free and gnotobiotic colonies, Interactions of colonies and/or chimeras, *Serratia* sp., Scouting, Rock-paper-scissors

## Abstract

**Background:**

Bacteria grown on semi-solid media can build two types of multicellular structures, depending on the circumstances. Bodies (colonies) arise when a single clone is grown axenically (germ-free), whereas multispecies chimeric consortia contain monoclonal microcolonies of participants. Growth of an axenic colony, mutual interactions of colonies, and negotiation of the morphospace in consortial ecosystems are results of intricate regulatory and metabolic networks. Multicellular structures developed by *Serratia* sp. are characteristically shaped and colored, forming patterns that reflect their growth conditions (in particular medium composition and the presence of other bacteria).

**Results:**

Building on our previous work, we developed a model system for studying ontogeny of multicellular bacterial structures formed by five *Serratia* sp. morphotypes of two species grown in either "germ-free" or "gnotobiotic" settings (i.e. in the presence of bacteria of other conspecific morphotype, other *Serratia* species, or *E. coli*). Monoclonal bodies show regular and reproducible macroscopic appearance of the colony, as well as microscopic pattern of its growing margin. Standard development can be modified in a characteristic and reproducible manner in close vicinity of other bacterial structures (or in the presence of their products). Encounters of colonies with neighbors of a different morphotype or species reveal relationships of dominance, cooperation, or submission; multiple interactions can be summarized in "rock – paper – scissors" network of interrelationships. Chimerical (mixed) plantings consisting of two morphotypes usually produced a “consortium” whose structure is consistent with the model derived from interaction patterns observed in colonies.

**Conclusions:**

Our results suggest that development of a bacterial colony can be considered analogous to embryogenesis in animals, plants, or fungi: to proceed, early stages require thorough insulation from the rest of the biosphere. Only later, the newly developing body gets connected to the ecological interactions in the biosphere. Mixed “anlagen” cannot accomplish the first, germ-free phase of development; hence, they will result in the consortium of small colonies. To map early development and subsequent interactions with the rest of the biospheric web, simplified gnotobiotic systems described here may turn to be of general use, complementing similar studies on developing multicellular eukaryots under germ-free or gnotobiotic conditions.

## Background

All living beings find themselves embedded in a complicated and fluid network of ecological (symbiotic) interdependencies. Ontogeny, i.e. buildup of a multicellular, species-specific body, may represent an exception: early stages of embryonic development typically require massive shielding against the influences of biospheric web. Thus, animals and plants go to great pains to ensure sterile conditions for their embryos; even fungi, champions of web-dwelling who spend most of their life without apparent body patterning, produce a special, protected cocoon (“embryo”) whenever they decide to produce fruiting bodies – mushrooms typical of their kin. Bacteria, typical dwellers of multi-species consortia, are allowed to build such species-specific bodies only at rare occasions when they can claim suitable germ-free environment (like freshly ruptured fruits, loafs of bread, surface of milk, etc.). Only then we can admire their creativity in building macroscopic, species-specific bodies (colonies). Bacterial axenic, i.e. germ-free growth on solid media reveals many paraphernalia of their ontogenetic potential (e.g., [1-5]).

Subsequent coupling of the developing embryo to the biospheric web often requires a thorough coordination. For example, all animals populate their bowels with a microbiome consisting of hundreds of microbial species (e.g., [6]). Some animals even require such cooperation for their proper organogenesis; as in the squid-*Vibrio* interplay in the development of light organ [7], or in mycetome of insects [8]. In plants, mycorrhiza or legume-*Rhizobium* symbioses [9,10] belong among paradigmatic examples. To disentangle such complicated interactions, development under germ-free or gnotobiotic conditions (involving two or at most a small number of interacting species) is often of a great help. Similarly, a “gnotobiotic” state, i.e. controlled development of bacterial colony in the presence of other bacterial bodies, may reveal rules and factors of cross-species interactions that otherwise remain obscured by their usual – consortial – way of life.

Bacterial colonies offer another advantage: Whereas most “typical” multicellular organisms steer their development towards a body capable of reproduction, for most bacteria building a multicellular body is not *the* precondition for maintaining the lineage. If, in spite of the fact, they do not end in topsy-turvy assemblages of cells, structured multicellular bodies must help somehow in marking out and holding their spatial and temporal claims. Hence, whenever freed from the grip of ecological demands in the consortium, they orient their full creative potential towards a single multicellular body.

Putting such bodies into contact with similar bodies – of siblings, of other strains or other species – may reveal some basic rules of bacterial interactions that are valid not only for such gnotobiotic situation on the dish, but also in natural consortia. In a similar way, chimeric “colonies” started by a mixture of different bacterial lineages, may shed light to “colonizing processes” that take place in incomparably more structured, multispecies ecosystems intangible experimentally. Such an approach may be more informative than is the usual study of growing homogenous suspension cultures. In fact, trends towards developing multicellular structured bodies (colonies, films, coatings, fouls, etc…) fail *only* in well-mixed suspension cultures: it seems that the planktonic way of living is rather an extreme, an exception from usual life strategies of most bacteria (e.g. [11]). Yet, most information concerning bacterial communication comes from suspension cultures i.e. unstructured mass (e.g. [12,13] for quorum sensing; [14] for signaling via antibiotics); but see works on intricate networks of quorum regulations in *Serratia* biofilms [15-17]. “Morphogenetic” data on colonies were mostly obtained under stress conditions (as is the presence of antibiotics, phages, etc.), and the goal of such experiments was primarily diagnostical, not aimed to study developmental processes as such. Many authors therefore consider results obtained from suspensions to be more representative, more “true” than those obtained on bacterial bodies.

In contrast, in this paper we focused on revealing steps towards a simple ecology on the Petri dish: how multicellular bacterial structures (colonies or chimeras) feel the self and the nonself, and how they react to the presence of the others. We draw from earlier works on bacterial colonies [4,5,18,19], but above all from our previous studies on developing *Serratia* colonies [3,20]. Thanks to color and plastic patterning, their development is easy to follow, without a need of artificial molecular or genetic markers. Moreover, our morphotypes show a finite colony growth, i.e. the whole development takes place in a limited area, and the markers of youth, prime, and senescence are readily apparent. Due to relative “simplicity” of their “embryogenesis”, colonies offer insights into strategy of establishing morphogenetic fields, evaluating the quality and amount of space available, and reacting to bodies occurring in the immediate neighborhood – both conspecific (i.e. in axenic cultures) or heterospecific/heterotypic (i.e. under gnotobiotic settings).

We further utilized a gnotobiotic approach in the study of bacterial consortia. We believe that simple chimeric communities, such as those developed in the present work, will provide a pathway towards understanding behavior of the utmost important ecosystems on the Earth – those of the prokaryotes (e.g. [21]).

We designed our study with the assumption that bacterial way of life is primarily multicellular [22]: they form a body that comes to existence through a sequence of elaborated, species-specific morphogenetic processes, in a given environment. (It means that we shall not consider such phenomena as flocculation, even if we admit that even such aggregates may bring a selective advantage in comparison to planktonic way of life; see, e.g., [23,24]). Depending on initial setting, bacteria can develop two kinds of multicellular existence: (1) Axenic, “germ-free” clonal growth from one cell or from a group of cells of the same kin, leading to a colony or a swarm (often with a fruiting body). Such colonies then command a plethora of strategies how to implement their fitness towards neighboring bodies. (2) When the conditions do not allow an axenic start, due either to simple crowding, or to the presence of competing clones and species, the body-building strategy will change towards small colonies in close contact that establish consortia elaborately interconnected with other dwellers of the community (e.g. stromatolites, plaques, or mats; [25,26]). An interesting phenomenon occurs when the edge of such a chimera grows into free substrate: often it will radiate rungs of monoclonal material; this phenomenon is apparent even if the chimerical body contains close relatives [3,27,28].

**Figure 1:**
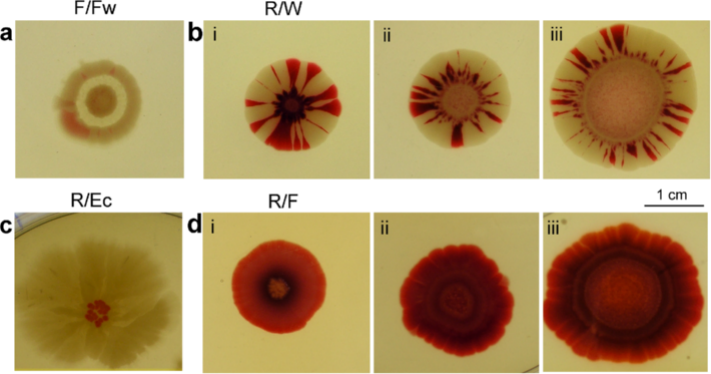
**Single colony morphotypes, on NAG medium. a ***S. rubidaea ***R** and **W** forms; **b ***S. marcescens ***F**, **Fw**, and **M** forms*;* and **c *E. coli****.* Left: colony appearance at maturity (7–9 days), with schemes of colony cross-sections. All *Serratia* colonies show terminate growth: final diameter is about 15 mm in **F**, **Fw**, and **M**, 20 mm in **R** and **W**. Right: development of colony margins at days indicated (free agar is at the right).

## Results

### “Standard” development of solitary colony morphotypes

For our study, we selected two mutually related stable morphotypes of *Serratia rubidaea* (R and W) and three morphotypes of *S. marcescens* (F, Fw, and M). A common laboratory strain of *E. coli* was included in some gnotobiological experiments.

**Figure 2:**
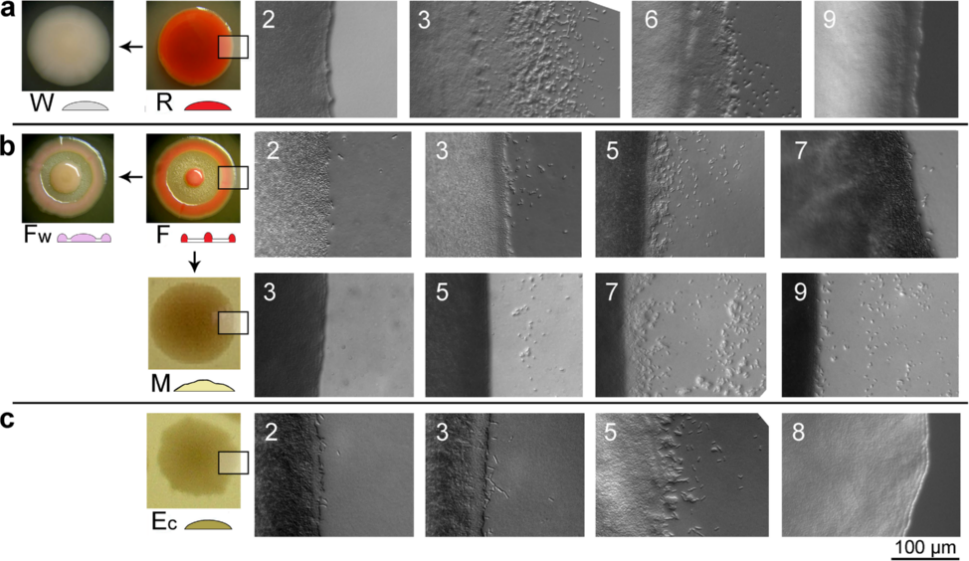
**Role of external factors in colony patterning.a** Effect of temperature: development at 27°C and 35°C, on NAG. **b, F** colonies, effect of transfer from 35°C to 27°C. Diameters of colonies in **a** and **b** are normalized: real diameters grow from 1 mm at day 1 to 15 mm at day 7 for **F** and **Fw**, or 20 mm for **R** and **W**). **c** Effect of cultivation on different media on the appearance (day 7) of **F** colonies (sugars or alcohols added as nutrients; PEG as an osmotic). NA – nutrient agar, TN – tryptone. **d** Effect of delayed glucose addition on **F** colonies planted on NA (day 12). Note the absence of glucose effect after 3 days on NA.

Figure 1 shows the typical adult appearances of all morphotypes growing as single bodies on NAG substrate (nutrition agar with added 27 mM glucose, 27°C), with the time-course of colony margin development shown at higher resolution (for corresponding macroscopic appearance of developing colonies see Figure 2a). *Serratia rubidaea* colonies (Figure 1a), sown at a mutual distance of minimally 20 mm, grow as smooth, glossy, radially symmetrical red colonies (**R**) that frequently give rise to a stable colorless variant **W** (white). Our *S. marcescens* strain gives, on the same medium, a stable, rimmed morphotype **F** (“fountain”) that also produced a stable white variant, **Fw** (Figure 1b, see also [20]). Except of color, the behavior of white variants **W** and **Fw** were interchangeable with their colored parents, **R** and **F**, respectively; that gave us advantages in further experiments involving colony interactions.

**Figure 3:**
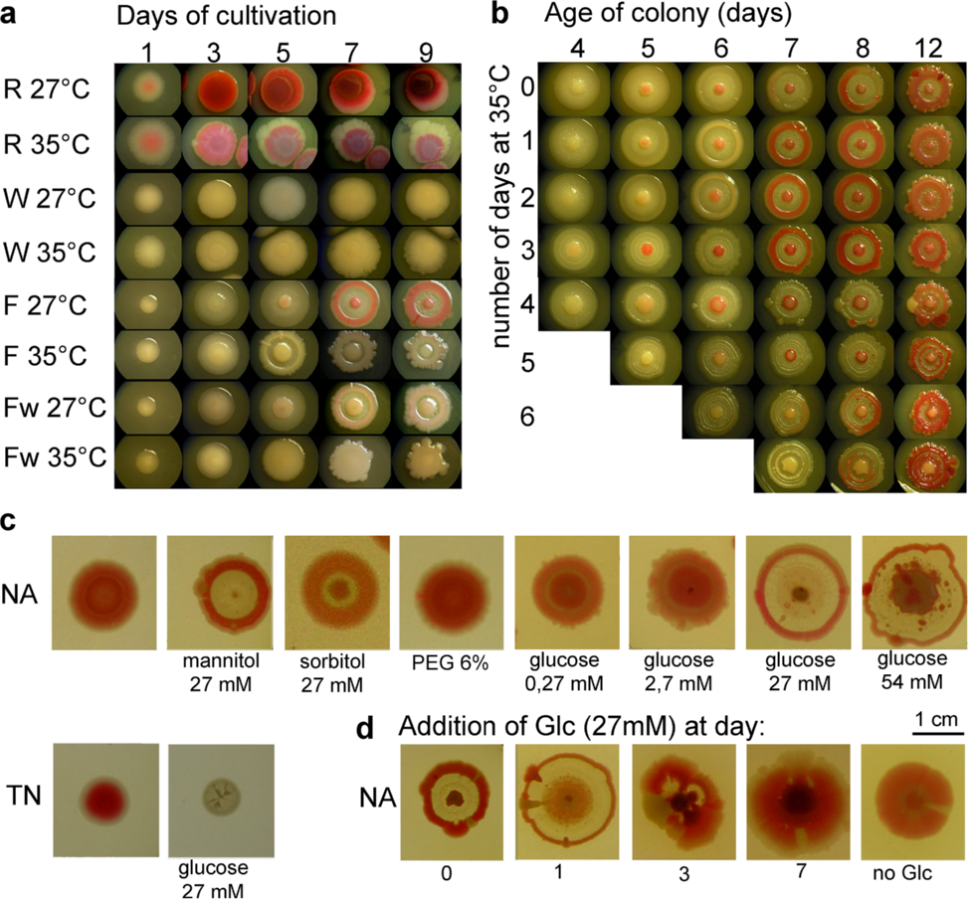
**Modification of F colony structure by neighboring baterial bodies. a** Formation of **X** structures of the **F** morphotype in the vicinity of **non-F** maculae (day 10) on media with (i-iii) and without (iv) glucose (NA *vs.* NAG); **b** Cross-section diagram of **X** structure and the microscopic pattern of its margin.

The fifth clone, **M**, was selected upon long-term cultivation of the **F** morphotype on liquid minimal medium (MM). On the rich medium NAG (or NA) it produces white optically undifferentiated, rimless colonies (Figure 1b). Finally, the appearance of our strain of *Escherichia coli* is shown in Figure 1c.

As to the microscopic features, the macroscopically smooth **R** (or **W**) colonies (*S. rubidaea*) develop terrace-like layers of cells at the margin, as if the growth proceeded in waves of juxtaposed plies. The lowermost, and the quickest layer, however, has no clear-cut edge, and dispatches cohorts of freely moving cells (“scouts”) into the space beyond; the main body of the colony will grow into the area previously “investigated” by the scouts. With the arrest of growth in adult colonies, the scouting decreases and finally ceases (Figure 1a). In contrast, the rimmed **F** (or **Fw**) colonies of *S. marcescens* start with a fluffy verge, replaced by an edge of more solid appearance on day 3; terraces do not appear (Figure 1b). Again, from day 3 on, flocks of scouts travel beyond the edge into the free space around, to subside with maturation and cessation of growth. The adult **M** morphotype of *S. marcescens* (Figure 1b) differs from its parent (**F**) by a sharp margin, and delayed scouting (after day 5). Finally, Figure 1c shows development of an *E. coli* colony under identical conditions; colonies of this species also develop terraces on the margin, and send out scouts during vigorous colony growth.

### Developmental plasticity induced by varying culture conditions

It is important to stress that given morphotypes develop towards phenotypes described in Figure 1 only under strictly defined culture conditions (the extreme sensitivity of colony structure to cultivation protocols in *Bacillus* see also [1,29], in *S. cerevisiae*[30]). Different media and/or conditions will lead to different patterning (see below); we have investigated the effects of temperature and manipulations with media composition in more detail. Similarly, the presence of colonies of either *S. rubidaea* or *E. coli* in the vicinity leads to a switch of the **F** morphotype into a new structure (called below **X**, see Figure 3).

**Figure 4:**
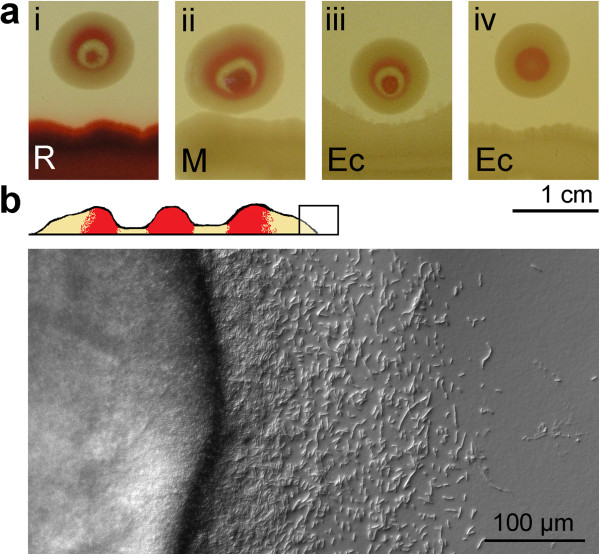
**Induction of growth of F colonies on minimal medium (MMA) by maculae: a R macula; b M macula; c *E. coli *macula.** (Day 7) Middle row: macroscopic appearance, top and bottom row – magnified details (see inserts the macroscopic structure). Note the smooth, non-interactive edges without scouts. **d** Helper colony of ***E. coli*** (arrow) in center of dense sowing of **F**. (Day 7). Bars: 1 cm in all macro-, 100 μm in all micro-photographs.

#### Effect of temperature

**R**, **W**, **F**, and **Fw** morphotypes were planted on NAG at three different temperatures: 27°C (standard development), 6°C, and 35°C. As expected, at low temperature the bacteria did not grow, albeit they survived for long periods and upon transfer to permissive conditions (27°C) resumed standard growth, after some lag (data not shown).

Cultivation at 35°C (Figure 2a) did not affect the final colony size, yet early phases of growth proceeded faster, and the colony patterning frequently deviated from the typical symmetry (especially in **F**, **Fw**); moreover, the coloration was lacking (**F**) or disrupted (**R**). Hence, higher temperature somewhat interfered with morphogenetic events. As shown in Figure 2b, the effect is, in **F** morphotype, fully reversible up to about the 3^rd^ day of cultivation at elevated temperature; older colonies transferred to 27°C did not attain the standard colony form in spite of striving towards it, as testified by the onset of coloration. Such a critical time threshold in 3^rd^ day is apparent also in connection with the effect of added glucose (see below, Figure 2d).

#### Effect of media

The standard appearance of the **F** phenotype (Figure 1b) was described for colonies grown on nutrient agar NA supplemented with 27 mM glucose (NAG). Replacement of glucose by sorbitol or mannitol at the same concentration allows for a “partial” F pattern. Lower glucose concentrations (0.27 or 2.7 mM) do not support standard patterning; higher concentration (54 mM) deforms the final pattern. Semi-defined medium of comparable composition (TN, or TN with added glucose) supports healthy growth of well-formed colonies, albeit with a patterning different from the phenotype grown on NAG. Finally, polyethylene glycol (PEG) added to NA in amount mimicking the osmotic load caused by 27 mM glucose did not promote the standard development (Figure 2c).

#### Effect of glucose addition during development

At various times after planting on NA, **F** colonies were “circumscribed” with glucose solution, to achieve its concentration, in the agar, in the range of about 27 mM in the immediate vicinity of the colony. As shown in Figure 2d, the older the colony, the more difficult for it to accomplish the standard appearance after glucose addition: after the 3^rd^ day the “struggle towards form” became distorted, and the inner (intermediate) ring did not appear at all (even if under normal condition it grows until 5^th^ day; see [3]).

All these effects of culture conditions are fully reversible in the sense that cell material taken from “atypical” colonies reverts to standard appearance when planted to NAG; thus, we are dealing with true developmental plasticity rather than selection of variants.

### Morphotype F: development in the presence of neighbors

As already reported, **F** colonies are very sensitive towards neighboring bodies on the dish. Closely planted **F** (or **Fw**, or **F** and **Fw**) colonies grow into a confluent colony with multiple centers and a common rim. An **F** macula will inhibit normal growth and patterning of **F** (or **Fw**) colonies growing in their vicinity, even when planted across a mechanical septum. Finally, heterospecific bodies (colonies or maculae of *S. rubidaea* or *E. coli*) were shown to induce formation of a new quality, a special pattern named **X** structure, characterized by an additional ring round the standard **F** colony [3,20].

Here we investigated the formation of **X** bodies in a closer detail (Figure 3; see also Figure 6a). First, we found that even the **M** clone (i.e. the rimless derivative of **F**) can induce the **X** structure in **F**. We also found that, in contrast to standard development, there is no critical period of induction: the **X** structure will appear also on an older, or even adult and non-growing **F** colony, if a **non-F** body is planted nearby. The characteristic patterning of the **X** structure is apparent also at the microscopic level, revealing a margin (devoid of terraces) and scouting pattern somewhat different from typical **F** (where scouting recedes by the time of maturation; compare Figure 1a and Figure 3b). It is remarkable (in the context of results discussed below), that the margin pattern is identical around the whole perimeter of the **X** structure (even if the structure macroscopically, as well as microscopically first appears on the site adjacent to the neighbor). Like in the previous cases, the transformation is developmental (i.e. not genetic), as the cell material taken from **X** will give, upon planting under standard conditions, rise to a typical **F** (or **Fw**) colony.

**Figure 5:**
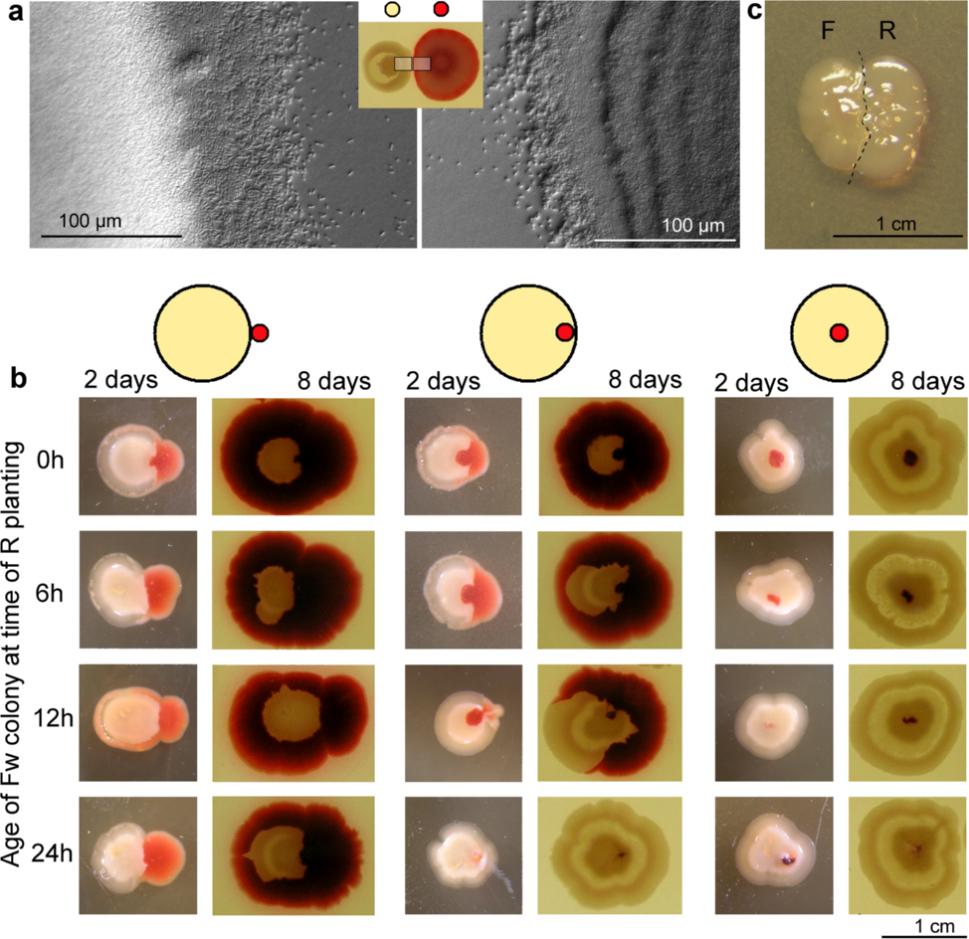
**Interaction of homospecific neighbor colonies. a R** colonies; **b F** colonies at two different distances; photos of adult colonies (Day 10). In micro-photographs (i-iv) only adjacent faces are shown; the distal faces of the colony are similar to fully developed controls shown in Figure 1a, b.

The induction of an **X** structure takes place also on NA (i.e. without glucose, Figure 3a, iv): it follows that the **F** morphotype can react by an **X** buildup regardless of its actual phenotype at the time of induction. The effect is exerted also when **F** is planted to the substrate previously conditioned by growth of any **non-F** body (not shown). Hence, the colony is receptive to the “make **X”** order under a great many of initial conditions and the X-inducing signal persists in the agar substrate.

#### Growth on minimal medium

On rich medium such as NAG we observe exigent structures and coloration in both *S. rubidaea* and *S. marcescens*; it was of interest to what extent, if at all, such patterns would develop on the minimal medium agar (MMA). **R** and **W** morphotypes (colonies or maculae), as well as our strain of ***E. coli***, grow readily on MMA, yielding, however, only white (occasionally faint pink in case of **R**), concentric colonies that do not allow distinguishing a given morphotype by its appearance (see Figure 11b). Moreover, of great interest is the *absence of scouts* and the *absence of marginal cascades* (Figure 4) in all types or developmental stages of growing bodies interacting with their neighbors (see below). Morphotypes **F** or **Fw** of *S. marcescens* do not grow on MMA, although they survive on it for weeks as an unstructured smear, and upon transfer to NAG commence growth towards standard **F** or **Fw** patterns. Only after prolonged efforts to habituate **F** cells in *liquid* minimal medium (MM), we succeeded to obtain a new stable morphotype, **M**, that gives white colonies on MMA; on NAG it grows towards smooth white colonies with elevated center (Figure 1b). What is important, F colonies behave towards the **M** macula as if it were **non-F** material: **M** induces **X** structure in **F** when grown on NAG (Figure 3a, ii.).

**Figure 6:**
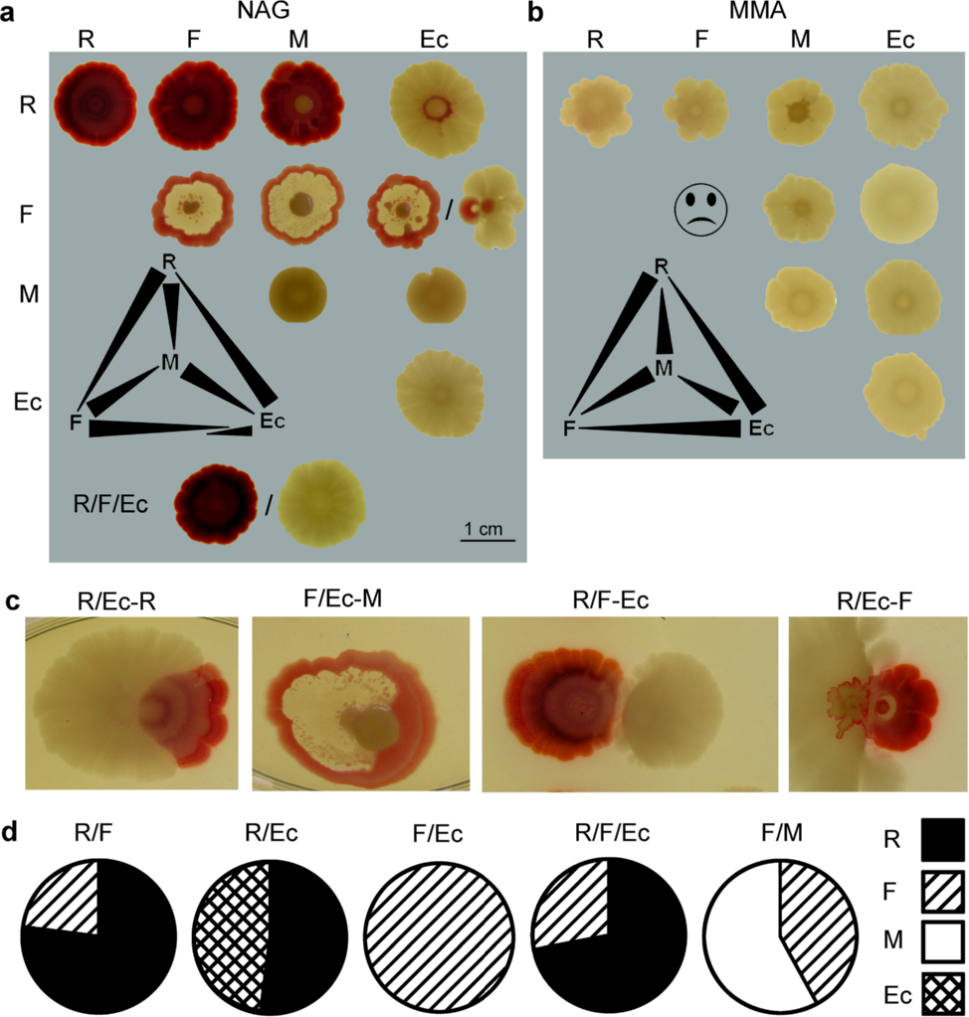
**Interactions of Fw and R colonies. a R** and **Fw** planted simultaneously at a distance of 10 mm - induction of **X** pattern in **Fw**; the microscopic image of the **X** periphery is uniform round the perimeter, whereas **R** scouts appear only in the interaction area (day 10). **b R** dotted to the vicinity or into **Fw** colonies (planted by dropping) of varying age (0–24 hours), photographed after 2 and 8 days of common growth. **c** Interaction of **F** and **R** on MMA, planting distance 3 mm; dashed line delineates the contours of both colonies (Day 7).

**Figure 7:**
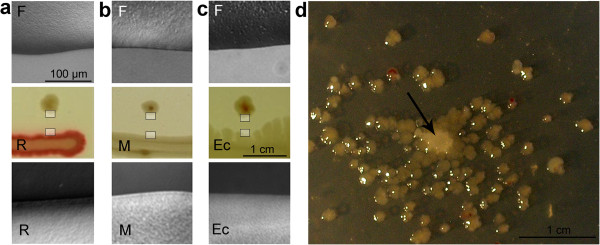
**Mutual sensing of F and *E. coli *colonies. a** At time 0, both partners were planted simultaneously at two different distances. Negative values: **F** planted to ***E. coli*** colonies one (−1) or two days old (−2). Positive value: ***E. coli*** planted to **F** colonies 2 and 6 days old (note the different magnification at lower left; arrow shows rudiment of ***E. coli***). Day 10 after planting ***E. coli*.** Micro-photographs taken from areas indicated. **b** Interaction on MMA, planting distance 3 mm; dashed line delineates the contours of both colonies. (Day 7).

Unexpectedly, however, the **F** morphotype is also able to grow on MMA when a “helper” in the form of a **non-F** body grows nearby (Figure 4): in such a case, it gives rise to small, smooth, white colonies that do not produce scouts or **X** structures.

**Figure 8:**
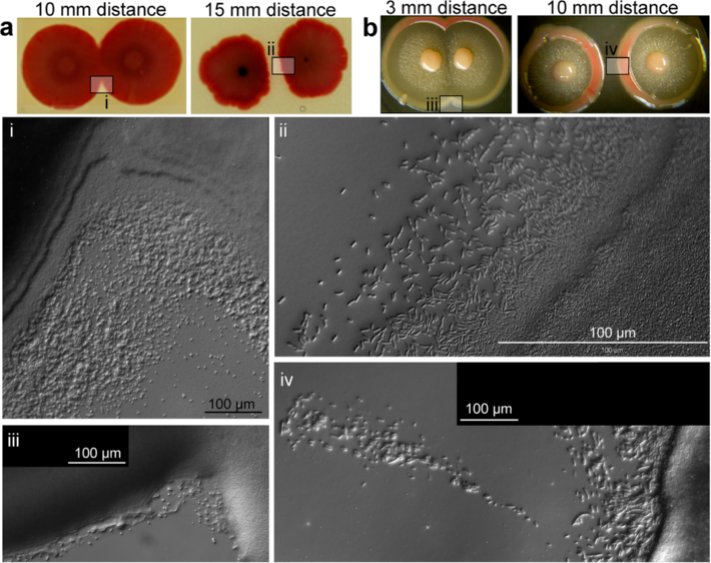
**Mutual sensing of R and *E. coli *colonies. a** At time 0, both partners were planted simultaneously 5 or 15 mm apart. Negative value: **R** planted to ***E. coli*** colony one day old. Positive value: ***E. coli*** planted to **R** colony 1 and 2 days old. Day 10 after planting ***E. coli***. Micro-photographs taken from areas indicated. **b** Interaction on MMA, planting distance 3 mm, dashed line delineates the contours of both colonies. (Day 7).

The adjacent edges of **non-F** macula and **F** colony, whether growing or not, appear sharp, and dispatch no scouts (Figure 4; compare below to Figures 5-8). There is also a difference in colony yield: An inoculum giving 50–100 colonies/cm^2^ on the NAG substrate, will give rise, on MMA, to only 5–10 colonies/cm^2^, and only at a distance of about 2 cm from the helper colony (Figure 4d).

**Figure 9:**
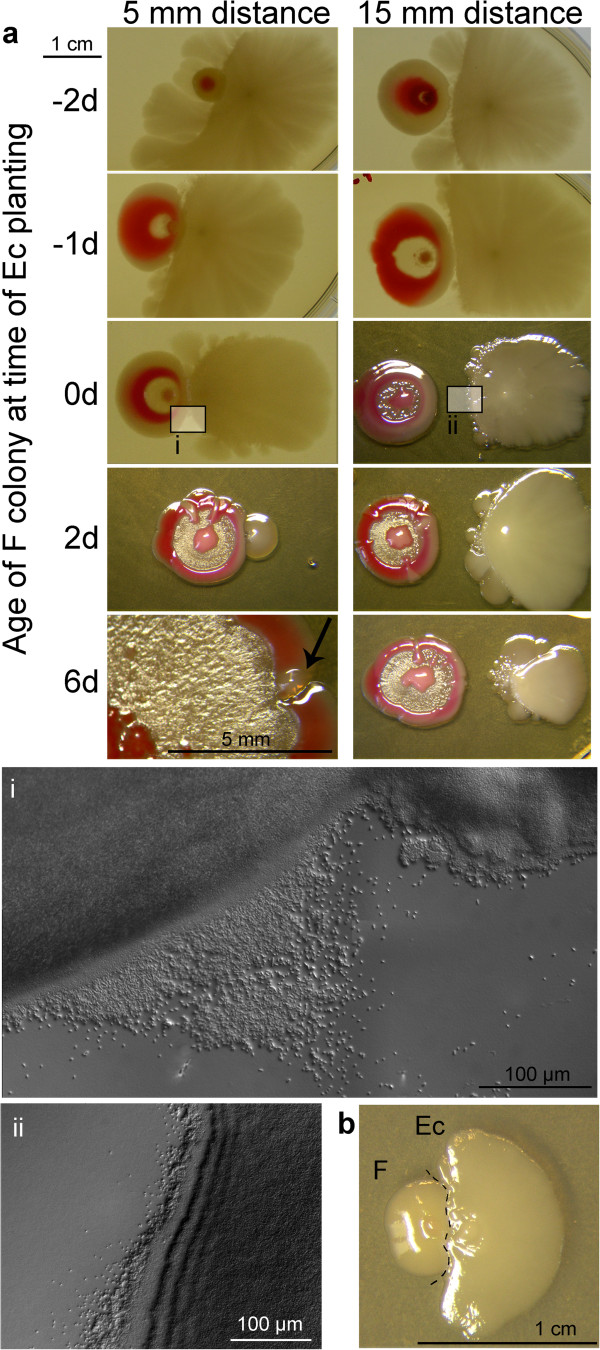
**Interactions of M bodies with neighbors. M** planted on **a** NAG or **b** MMA simultaneously into a close vicinity (2 mm) of **F**, **R**, or ***E. coli***. (Day 6).

**Figure 10:**
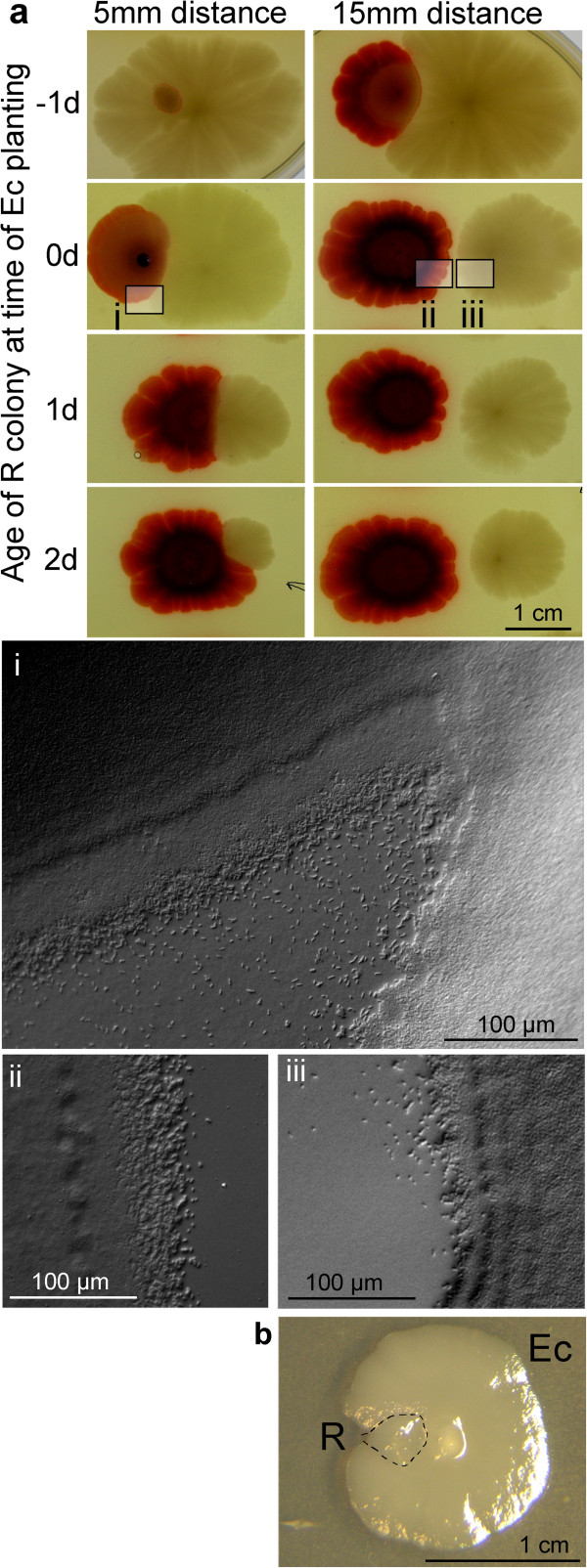
**Growth of chimeras. aF/Fw b R/W c R/Ec**, and **d R/Fw** chimera. Chimeras are either dropped, (**a-d,** i), or are spread to diameter of 5 (ii) or 14 mm (iii). Note the consortium in the planting area, with clonal outgrowths of both clones in case of **R/W**, or of the **R** clone only in case of **R/Fw** chimera.

Even old (10–14 days), non-growing, persisting **F** plants can be boosted to grow on MMA when a **non-F** macula (including also **M**) is added to the dish, or even when planted to a macula-conditioned agar (not shown). Cells taken from such boosted **F** colonies will not gain any (even transient) ability to grow independently on MMA; when planed to NAG, however, they give rise to normal **F** pattern.

Thus, the **F** morphotype might be dependent on some essential nutrient or signal present in NAG but not MMA; such a trigger diffusible in agar may be provided by the growing macula (non-growing **F** “macula”, i.e. a mass of non-growing **F** cells applied to the dish, having no effect), and survives in the medium for longer periods. Preliminary results (not shown) suggest that the case may not reside in basic nutrients. First, the *E. coli* 15 TAU strain (auxotrophic for arginine-thymine-uracil) does not grow on MMA even in the presence of helpers, or on a conditioned agar (it also cannot serve as a helper when, as in case of **F** above, a mass of non-growing cells is applied to the vicinity od **F,** on MMA). Second, the **F** morphotype will not resume growth on the MMA even if the substrate is supplemented with casamino acids (caseine hydrolysate with cysteine and tryptophan added).

### Mutual influencing of colony habitus

The ability of the **F** morphotype to develop towards a new pattern in the presence of heterotypic (i.e. **non-F**), neighbors instigated us to take a deeper look on the mutual interaction of our standard colony types.

#### Homotypic interactions R:R and F:F

Figure 5 shows the simplest configurations of two colonies of the same morphotype planted to close vicinity. Such colonies may come to a contact and even, in case of **F**, merge into a confluent colony; when planted further apart, they remain separated, albeit shape deformations occurred frequently (Figure 5a). The common feature of two approaching colonies is the presence of *scouting* bacteria beyond both adjacent (and approaching) colony edges – even in older colonies (10 days), when no such “freelancers” are observable in solitary colonies of comparable age (Figure 5 i-iv; compare to Figure 1a, b). In contrast, the distal side of an interacting colony showed no difference from the solitaires, i.e. no restoration of scouting occurred (not shown). Planting a young **R** colony to the vicinity of an old one (**R**, 3 weeks) aroused a new wave of scouting towards the new neighbor, in the old colony (not shown). The phenomenon is thus distinct from the induction of an **X** structure, where scouting reappears around the whole perimeter of the colony, accompanied by profound reshaping of the colony phenotype.

#### Heterospecific interactions: R and Fw

As expected, an **R** colony planted at a distance not allowing immediate overgrowth of its **Fw** neighbor (10 mm, Figure 6a) will induce formation of the **X** structure, which will resist any contact: both colonies persist as separate entities (as in Figure 3), with a typical colony pattern and scouting in the interaction zone.

Figure 6b summarizes situations when young (0–24 h, showing no typical structures) **Fw** colonies come into close contacts with a plant of **R**. The **Fw** colony will always be overgrown by **R** planted on its outer perimeter. The **Fw** material, however, maintains its identity in such a conjoint body, and its territory remains free of **R** cells. Note, in older colony, even an inclination towards the **X** structure – however it is belated and not able to avoid overgrowth by the neighbor.

Planting **R** to the inner perimeter of young **Fw** gives essentially the same picture: the **R** material breaks free and encircles the **Fw** if planting had occurred during the first hours of **Fw** development. After one day, however, the **R** material cannot “escape” any more, remains confined inside the **Fw** colony and does not grow (but survives). Finally, when planted into the center of **Fw**, the **R** material never resumes growth and remains encaged (but not killed) inside the **Fw** colony as a tiny island of foreign material.

All interactions on NA resemble to those observed on the rich medium NAG, including colony patterning (not shown). Different, however, is the interaction of both clones (planted 3 mm apart) on MMA: thanks to the helper function of **R**, both colonies grow to approximately equal size, and come to a close contact (Figure 6c). The **R** colony, however, will not encircle the **F** material (compare to Figure 6b).

#### *Heterospecific interactions: Fand* E. coli

The interaction of young **F** colonies with plants of ***E. coli*** (Figure 7a) is controlled by the **F** partner: if both partners planted simultaneously, ***E. coli*** avoids approaching **F** (see similar trend with the macula, Figure 3a, iii) and grows only at distal side. At the same time, the **F** colony develops an **X** structure induced by ***E. coli***. If planted to a distance of 15 mm, resulting adult partners maintain their scouts in the gap between them. Planting ***E. coli*** to older **F** colonies results in drastic inhibition of the growth of ***E. coli***.

Even more profound the effect is in closer plantings (5 mm apart): the ***E. coli*** plant will be “caught up”, and its growth inhibited proportionally to the age of **F** (Figure 7a); yet it survives and remains uncontaminated by **F** material, even in cases of strongest growth inhibition. The dominant role of **F** is even more profound when **F** material is planted to older ***E. coli*** colonies: even in such cases, the **F** body remains in control of events. Such an inhibition is not bound to the presence of living **F** cells: the **F-**conditioned agar has the same effect (not shown). The effect is identical at 35°C, i.e. the inhibition was not due to growth at temperature that may be considered suboptimal to of ***E. coli*** (not shown).

On the MMA medium (where the **F** material does not grow when alone), ***E. coli*** turns into a helper, a necessary precondition for the growth of **F** (Figure 7b, see also Figure 4a). Yet, the growth of ***E. coli*** becomes inhibited by the boosted **F** colony.

#### *Heterospecific interactions: R and* E. coli

As shown in Figure 10, ***E. coli*** is dominant only when the **R** material is planted simultaneously (or to an older) ***E. coli*** colony, and to a close vicinity (below 5 mm). In all other instances, both bodies are in control of their integrity: (i) they maintain a clear boundary when grown to confluence, and neither is able to overgrow the partner, or (ii) when planted farther apart, they respect the free space between the colonies. In comparison to previous situation (***E. coli*** and **F**), the ***E. coli*** colony, albeit inhibited, is not repulsed by the *Serratia* partner. Again, mutual contacts induce appearance of the scouting at adjacent faces of both colonies.

Interaction of both morphotypes on MMA leads to a dominant role of ***E. coli***: the **R** material is strongly inhibited (but survives) and becomes engulfed by readily growing material of ***E. coli*** (Figure 8b).

#### Interactions involving the M clone

Interactions of **M** colonies, planted simultaneously to a close vicinity (cca 2 mm) to heterospecific plants are shown in Figure 9. On the rich medium NAG (Figure 9a) no confluent colony appears with the “mother” **F** morphotype: instead, **M** was encircled by **F** (but surviving). On the other hand, **M** becomes encircled and inhibited by **R**, as is **F,** its maternal clone (see Figure 6b). Also in the third setting – **M** with ***E. coli*** – the repulsive effect on ***E. coli*** was similar to that observed in **F** (see Figure 7). On the MMA (Figure 9b), the **M** exerts the helper effect for **F**, yet the **F** colony remains small and unstructured. Interaction **M-R** reveals partners of equal strength on the minimal medium, whereas ***E. coli*** is retreating as on NAG.

**Figure 11:**
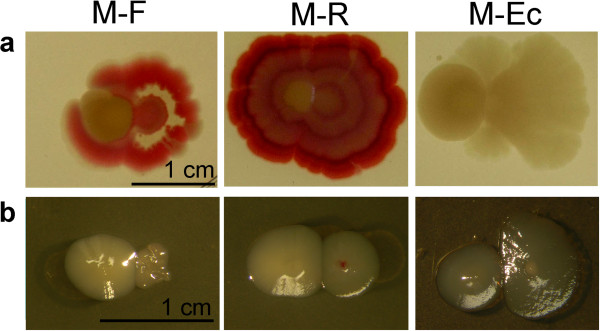
**Growth of chimeras - a summary.** Growth on NAG (**a**), or MMA (**b**) (white variants **W**, and **Fw** not included). Each matrix shows the appearance of possible combinations (see also Table 2), plus the ternary mix **R/F/*E. coli*** on NAG below. Tetrahedral schemes show dominance/submissivity relation for each combination; arrows widen towards the more dominant partner. **a** On NAG, **F**, **R**, and ***E. coli*** play the rock-paper-scissors game, and the same holds for the combination **M**, **R**, and ***E. coli***. Two remaining triangles show absolute dominance of **F** or **R** in particular settings **b** On MMA, ***E. coli*** and **M** dominate the field, whereas **F** is the absolute loser towards all partners. Smiley - no growth of **F** colonies. **c** Interactions of chimeras with colonies on NAG. (simultaneous planting to a distance of 5 mm, chimeras to the left, day 7). **d** Growth of suspension mixes in NBG - proportions of particular morphotype.

#### Binary interactions in liquid media

To investigate to which extend could the above-described phenomena explained by differential growth rate of individual clones, we investigated the growth of the studied morphotypes in liquid media NBG (identical, except for agar, with NAG).

Judged from doubling times Table 1 the **R** and **W** morphotypes should exert highest fitness in all interactions studied. Obviously, this is not a rule, and ecological interactions and mutual influencing enter the game in case of multicellular bodies growing on agar substrates (cf., e.g., the doubling times of **F** and ***E. coli*** in NBG, and the communication of their colonies in NAG). Inhibition of ***E. coli*** by **F** (Figure 7), massive overgrowth of **R** by ***E. coli*** (Figure 8), rapid circumspread of **R** along the margin of **F** (Figure 6), etc., all suggest the existence of interactions that appear at the level of multicellular structures, but cannot be discerned in suspension. Compare also two modes of overwhelming the neighbor: by “brute force”, as in case of ***E. coli*** towards **R** (Figure 8), or “strangling” (**R** towards **F**, Figure 6). The fact is even more noticeable in chimeras referred to below.

**Table 1:** Doubling times in liquid medium NBG (27°C)

**Morphotype**	**Doubling time**
	**[min] (F = 1)**
**F**	**64 (1.0)**
**Fw**	**73 (1.2)**
**M**	**58 (1.0)**
**R**	**38 (0.6)**
**W**	**37 (0.6)**
***E. coli***	**55 (0.9)**

### Chimeras

Chimerical assemblages result from planting not a single clone, but a mixture of two or more clones in a single plant (with equal contribution of all partners involved and with constant density of bacteria per unit of surface, Figure 10 and Figure 11). All combinations studied where both partners contributed to the result show a bipartite structure: (1) The area of planting (the navel of future pattern) hosts a consortium, i.e. a mix of small colonies of all members of the plant (see especially Figure 10). (2) Clonal outgrowths to the free space around the plant. This ruff is usually composed only from cells of a single morphotype, however, in cases when both partners are of equal “strength”, alternating wedges of both clones appear in the ruff (Figure 10a, b). The thickness of the ruff is essentially constant, independent on the diameter of the navel, and corresponding to the radius of single colony of particular cell material.

On NAG (Figure 11a), the only exception from the pattern is chimeras containing ***E. coli*** in combination with **F** and **M**. In such cases, ***E. coli*** was eliminated below the level of detection (no colonies out of about 1000 CFU per experiment), and a normal colony will result. Only occasionally ***E. coli*** manages control of the ruff, see below. Finally, a plant containing a mix of three morphotypes (Figure 11a) – **F:R:*E. coli*** (1:1:1) – led to two alternative outcomes. In most cases, the ruff consisted of **R** morphotype only, with the mixture of **R** and **F** in the central disk, with ***E. coli*** below the level of detection. Occasionally, however, as already observed in case of **F/*E. coli*** chimeras, the ***E. coli*** cells managed to outgrow to the periphery and control it, leaving a mixture of **R** and **F** in the central disk. In the disk, however, ***E. coli*** was always under the detection level, even in cases when the colony was started by a mixture **R:F:*E. coli*** 1:1:10 (not shown). The outcomes depend probably on how the mix escapes from the initial metastable state: (1) either **F** cells are able to keep at bay the ***E. coli*** population for a while, and both later get overgrown by **R** (compare to Figure 6b, Figure 7a); or (2) ***E. coli*** managed to acquire the control of periphery and did not let its partners grow out from the center.

On MMA, all chimeras (and colonies) have an almost uniform appearance, with a concave center, and white, broad ruff (Figure 11b); they are white, sometimes slightly pink when containing R cells. The exception is the **F** morphotype that, without helper, does not grow at all; chimeras **F/R**, **F/M** and **F/*E. coli*** eliminate **F** material below the detection limit; technically speaking, they build ordinary colonies.

All outcomes of chimerical growth on agar substrates are summarized in Table 2 and in Figure 11.

**Table 2:** Composition of central and peripheral areas of chimerical bodies

**Medium:**	**NAG**		**MMA**	
**Position:**	**center**	**ruff**	**center**	**ruff**
**R/M**	R + M	R	R + M	R < M
**R/Ec**	R + Ec	Ec	Ec > R	Ec
**F/M**	M + F	F	M	M
**F/Ec**	F	F/Ec > F	Ec	Ec
**M/Ec**	M	M	M	Ec
**F/R/Ec**	R + F	R/Ec	ND	ND

#### Mixed suspensions in liquid medium NBG

In order to test the possibility that the behavior of chimeras on the substrate is primarily deducible from the growth rates of partners, chimeric suspensions containing 1:1 inocula of partners, were grown also in the nutrient medium NBG (an equivalent of NAG, except the absence of agar). Figure 11d shows quantitative ratios of some combinations 24 h after inoculation.

Some results are in congruence with observations on chimerical bodies on NAG, i.e. **R** is dominant over **F**, and **F** dominates over ***E. coli***; in this case, however, **F** dominates absolutely, without rare cases of ***E. coli*** overgrowth. Similar is the dominance of **M** over ***E. coli*** (not shown). The proportions of **R/F/*E. coli*** in principle also match the situation observed on agar. The mixture **R/*E. coli***, however, with equal representation of both types, differs markedly from chimeras where ***E. coli*** always outcompetes **R** and confines it in the center of body. Mixtures **F/M** and **R/M** (not shown) grow at roughly similar rates, i.e. of no sign inhibition of **M** by **F** as observed on NAG.

#### Chimera vs. colony

The interaction of chimerical bodies with single-clone colonies (Figure 11c) planted simultaneously at 5 mm distance depends usually on what material is contained in the chimera’s ruff – essentially the interaction follows patterns shown in Figures 5–9 (such a typical case is the interaction of **R/*E. coli*** with **R** and **F/*E. coli*** with **M**). Some exceptions, however, deserve attention: In case of **R/F** chimera interacting with ***E. coli*** the result was not the chimera overgrown by ***E. coli*** (as in **R-*E. coli*** interaction. Figure 8a), but ***E. coli*** was effectively repelled, obviously thanks to the **F** material residing in the center of the chimera. Also interaction of **R/*E. coli*** chimera with the **F** body led, as expected, to an inhibition of ***E. coli*** by the **F** neighbor; this, however, enabled the **R** material to escape to the periphery and to overgrow the **F** neighbor.

#### Summary on chimeras

The outcome of chimerical interactions on both NAG and MMA substrates can be summarized by 4 schemes of interactions (triangular schemes in Figure 11a, b; for simplicity, the white derivates **W** and **Fw** are not included – they behave analogously to their parents, **R** and **F).**

Interactions, on NAG, in different settings, reveal a “rock – paper – scissors” relationship for two of four possible ternary settings: **R**, **F**, or ***E. coli*** and **M**, **R**, and ***E. coli*** (Figure 11a, scheme). In two remaining ternary combinations, **M** is always a loser (cf. also Table 2).

The situation is different on MMA, where ***E. coli*** always wins the contest in chimeras, whereas **F** is an absolute loser (Figure 11b, scheme): we are rather confronted with a hierarchy ***E. coli ≈*** **M > R > F**. The only exception to such a “pecking order” on MMA is not in chimeras but in colony interactions: if **M** or **F** (plus helper) get a chance to establish a colony, they take control over the in-growing ***E. coli*** in a way similar to that on NAG (Figure 7b).

## Discussion

We present here a simple system allowing study of bacterial development in two regimes of growth – germ free (axenic), or gnotobiotic. As mentioned in the Introduction, we draw inspiration from attempts to reduce extreme complexity of multispecies cohabitations from experiments with germ-free multicellular eukaryotes (mostly animals, or humans with inborn defects of immunity, but also plants) or gnotobiotic organisms where such a complexity was reduced to an interaction of two, or small number, of players.

### Germ-free development

Formation of multicellular bodies is facultative in bacteria: they easily survive and multiply without multicellularity, thus they can abound with much richer repertoire of creativity, without endangering further propagation of the lineage. Bacterial colonies, then, may provide some cues to the nature of multicellularity. Moreover, growth of a colony is a complex process specific for a given lineage, and specifically modulated by environmental conditions (neighbors, nutrients, spatial settings, an array of signals, etc.). We chose five easily distinguishable morphotypes belonging to two *Serratia* species; the sixth, “outgroup”, morphotype was a domesticated strain of *E. coli.*

It deserves a notice that our morphotypes seem to resist domestification, i.e. gradual loss of structural refinements when grown under laboratory conditions commonly observed in microorganisms [1,31]. What also deserves a comment is the fact that the way of initiating a colony has little, if any, effect on the resulting body building. The same pattern can be grown from a single cell, from big amount (millions) of cells planted to a limited area as a dense homogenous suspension, or even from a chunk of material from the donor colony. Provided the area of planting is small, the cells can coordinate their behavior, “make wise decisions and act upon them“(B. McClintock, The Nobel lecture, 1983). Regulatory embryos of metazoans provide another example of such a potential.

With our array of easily distinguishable morphotypes, we were able to proceed from “germ-free” colonies towards gnotobiotic colony interactions – either with conspecifics, or with heterospecific bodies. We believe that such arrangement may provide a promising tool for future study of microbial communication at the level of structured entities. Similarly, study of chimerical bodies introduced in our works may reveal rules controlling self-structuration of the bacterial body and/or multispecies community. Moreover, our hypothesis of two-phase formation of multicellular body (e.g. axenic and cross-talk stages) can be easily tested on bacterial bodies that are not constrained by the need of producing special reproductive structures (organs).

### Gnotobiotic interactions of clonal bodies

Perceiving the neighbors and interacting with them is one of the most natural conditions of all dwellers in the biosphere; often new qualities (shapes and properties) may appear as a consequence of such an encounter (for review, see [32]). Colonies growing on an agar plate provide a simplified model revealing some basic rules of such interactions [33].

In our model, a bacterial plant (be it a single cell or a clump of cells of a given morphotype) needs about 3 days to establish its “self”, to become a genuine multicellular body. During this initial period, its development may be readily deviated by external stimuli (Figure 2), or the presence of other bodies in its vicinity (Figures 3,9). Colonies of the same kin may even merge at this early stage of development (confluent colonies as reported by [20]), reminding early embryos of, e.g., of mammals.

In later stages of their development, colonies maintain their integrity even in inevitable close encounters, preferring a channel of free space between them, sometimes even “guarded” by advanced scouts; conspicuous is, in this respect, the “immune reaction” of rimmed colonies **(F, Fw)** that develop a specific “**X**” structure in the vicinity of rimless bodies (see also [3]). Even more accentuated such interactions become when colonies of different age grow to a close contact or are artificially forced to it – with the whole array of reactions such as breaking away from the neighbor, overgrowing it, “strangling” it, changing body pattern, changing the character of scouting, etc. (Figures 6,9). The roles of scouts remain enigmatic for the time being – albeit they may seem obvious candidates for mediators of short-distance interactions), because similar reactions of bodies do take place also on the minimal substrate (MMA) where we did not observe any scouting. What are they for, if obviously colonies can easily do without them?

Colonies on MMA appear as if underdeveloped: no coloration, no patterning, and no scouts. In this respects, they resemble very young colonies planted on NAG – as if the minimal medium impeded the transition from the juvenile phase into phase of growth and ornamentation (which would require scouts). Growth would, however, continue (as in experiments with higher temperatures, Figure 2), and the result is an “overgrown youngster”. Such a speculation may help to explain behavior on MMA, yet does not help explaining the *very* role of scouts in “full-blooded” development on NAG.

The ability to distinguish between self and non-self may represent one of the preconditions for consortial (or multi-species) way of life. The **X** structure, then, may represent such a reaction of **F** to the presence of foreign clones. Swarms of *Proteus mirabilis* (growing on solid media) display a similar behavior: whereas two swarms belonging to the same line will merge when grow towards each other, swarms of two different lines will maintain a demarcation line dividing both swarms [34]. The phenomenon is readily used in epidemiology, for diagnostics of different strains of *Proteus*. The mutual inhibition is communicated by secretion (and sensing) of a great array of signaling proteins – proticins [35]; similar system was described in *Pseudomonas aeruginosa*[36] Transforming *P. vulgaris* strain by a proticin from *P. mirabilis* leads to abolishment of mutual inhibition [37]. Yet, our observation of incompatibility even between isogenic strains (**R:R**, or **F:F**, see Figure 8) needs a more parsimonious explanation than rapid mutation of putative pheromone genes. As suggested by [38,39]), if an identical signal is produced by approaching siblings, it may lead to a quick surpassing of the quorum threshold in the furrow between them – this will lead to the inhibition of growth in that direction.

As a rule, we can recognize a “rock – paper – scissors” interplay between colonies belonging to three groups: (1) rimmed morphotypes **F**, **Fw**; (2) rimless morphotypes **R**, **W**; and (3) ***E. coli***, as summarized in Figures 5,9. The morphotype **M** has a somewhat intermediary position. Hence, even such a reduced, model “ecosystem”, will establish relations of dominance, cooperation, or subordination according to overall context. For the time being we were able to prove that the induction of **X** structure is the matter of a signal diffusing, and persisting, in the agar substrate (see also [3]).

A similar situation was already described described by Kerr *et al.*[40]: the authors cultivated three strains of *E. coli*, one producing colicine and being resistant to it, the second not producing but resistant (i.e. growing in the presence of colicine), and the third sensitive (i.e. killed in the presence of colicine). The authors interpret the results in neoDarwinian frames: The synthesizer will always overgrow the sensitive strain. Because of the cost of colicine synthesis, the resistant wins the contest with the synthesizer. As resistance itself represents extra cost, the sensitive strain will win over the resistant, but is a loser in a contest with the producer (see also [41]).

The harsh behavior of our *S. marcescens clones* (**F**, **Fw**, **M**) against ***E. coli*** might be explained as a relation producer – sensitive. For example Fuller & Horton [42] described production, by *S. marcescens*, of a factor dubbed marcescin, resembling in its effect to colicins. In such a schema, **F** would be in a role of the producer of the repellent; **R** would be resistant towards it – and therefore overgrowing the **F**, but at the same time sensitive to ***E. coli***. We suspect, however, that the situation is more complicated and more factors are in the game.

The phenomenon of cooperation comes to the fore even more with “helpers”: on the minimal medium, the morphotype **F** can grow only in the presence of rimless morphotypes or ***E. coli***, as it is dependent on – at present unknown – nutrient or signal secreted to the substrate by the helper. Yet, as soon as helped, **F** can exert its “powers” towards the neighbors: even if **F** colonies can grow only thanks to the ***E. coli*** plant in the middle, the same plant will later be strongly inhibited by colonies it supports (Figure 7b). Even more illustrative is the interaction of the trio **R**, **F**, and ***E. coli***. The R/E.coli chimera (normally the growth of **R** suppressed) in the vicinity of **F**, the **F** will keep ***E. coli*** at bay (as in Fig. 9), which enables **R** to grow and, in turn, overgrow and suppress the **F** (Figure 11c). All such interactions may be considered as paradigmatic for much more complicated ecosystems of natural microbial consortia.

### Chimeras

The dominance/subordination rules as observed above for colony encounters more or less fit also for chimeric growths; i.e. they are not explainable from the growth rates of particular morphotypes involved, as observed in suspensions (Graph in Figure 11d). Which of the partners will prevail will often depend by rock – paper – scissors rules – as described for single colonies. This is not surprising when we take into account that the chimera represents a model gnotobiotic microbial ecosystem. The dense initial mixed suspension on the area of planting is not able to negotiate the rules how to build the final body: Compare to situation with planting axenic cultures, where even very dense suspension establish a full-fetched colony indistinguishable from that growing from a single colony. An exception is “chimeras” where one of partners is completely eliminated, and the “winner” continues in building an ordinary colony (Table 2, Figure 11). Hence, in cases when all strains present in the mix survive, the planting area represents not the center of a colony, but a gnotobiotic ecosystem containing a nebula of very small colonies. An organized outgrowth from this navel will build the external circle composed of a single morphotype, or containing alternative wedges, each of a single morphotype. A chimera, thus, does not represent a body, but a consortium of bodies, even in simple gnotobiotic settings; only the clonal outgrowths into the free space may be compared to genuine colonies, albeit “one-dimensional”.

It deserves attention that even closely related sister clones **F-Fw** and **R-W** will not cooperate in building a single colony upon chimeric planting: Especially conspicuous is the “chrysanthemum” appearance of **R/W** chimeras (Figure 10). The finding is not new. Korolev *et al.*[28] working with a different pair of strains, argue that cells that happen to appear on the margin of the plant, will establish cooperating groups of this of that origin. They take over a corresponding part of the circumference and grow out of it as monoclonal, one-dimensional colonies – hence the “petals” of the chrysanthemum. Remarkably – in quoted studies as well as in our results – outgrowing “petals” grow to similar length, independently on the diameter of the planted navel. Again, the rock-paper-scissors rules (Figure 11) will mostly predict the outcome of the growth; the rest of interactions being hierarchical.

The mutual behavior of strains is more or less similar on both substrates tested, rich (NAG) and minimal (MMA); the only expected exception is the submissive role of **F** on MMA whose growth is dependent on the presence of helpers. It is conspicuous that the role of **F** is fully taken by its daughter morphotype **M**. As already mentioned above, the behavior of particular strains in liquid media provides no guide for predicting their behavior on solid substrates: the two kinds of media represent to a great extent alternative, and incompatible, strategies of growth.

#### Why multicellular bacteria?

If we take axenic bacterial colonies as analogues of clonal body of multicellular eukaryots, two problems will come out immediately: the *objective* of building such a body, and the *high plasticity* of bacterial ontogenies. As far as we know, colonies of *Serratia* never produce reproductive organs: they can safeguard their propagation without any demanding, and coordinated, activity of colony building. Why, then, do they go into the trouble with elaborate microscopic filigree of terraces and scouts, and even macroscopic patterning and ornamentation? The answer may lie in physiological division of labor [4] and perhaps even “histological” differences across the colony.

Besides plastic responses, bacteria can – reversibly or irreversibly – diversify also *genetically* into different morphotypes, depending on conditions like those mentioned above. In *Paenibacillus* repeated and heritable switches between different morphotypes are induced by the density of agar [43-45]. Genetic differentiation was also often described in suspension cultures. For example a clone of *Pseudomoas aeruginosa* differentiated quickly and apparently purposelessly into multiple genetic variants [46]. The authors ascribe the phenomenon to an “insurance effect” preparing the lineage to conditions that may set in the future. A similar effect in *Serratia* is believed to play a role in colonization of new niches [47]. Finally, a clonal population may break into different specialized clones evoked by metabolic demands [48,49] or antibiotic pressure [50].

However, since our clones were genetically stable in respect to the observed characteristics, and since all morphogenetic variation was found to be fully reversible, we can exclude such genetic switches, as well participation of phages, plasmids, transposons or similar elements, in our model and ascribe all variations observed (like colony patterning, scouting, or response to neighbors and environmental cues) solely to phenotypic plasticity.

## **Conclusions**

Multicellular bacterial models (colonies) match their eukaryotic counterparts (animals, plants, fungi) in areas of research classically focused only to eukaryotes:

1. Axenic (“germ-free”) and gnotobiotic settings are easy to establish, and interactions within the body, as well as between different bodies (of the same, or different lineages) can be studied to minute details. Such studies can be carried out on developing, fully formed or mixed assemblages of colonies that can be brought into defined spatial and temporal configurations. An additional advantage of the bacterial model is its independence on mature individuals that are able to produce germs (sexually or asexually), i.e. the range of full-formed phenotypes is much greater and can be influenced towards many ends (plasticity).

2. Ontogenesis of a colony (starting either from a single cell or from an assemblage of cells), similarly to the development of multicellular eukaryotic bodies, proceeds in two stages: the first stage must be thoroughly insulated from the rest of the biosphere and relies to intrinsic settings of the developing germ; in the second stage, the germ establishes its bounds with its environment, and plastically reacts to outside cues. In chimeric assemblages where the first phase is wrecked, the mix is unable to establish germ(s) and proceed towards a colony, and develops toward a simple bacterial consortium. Such an “ecosystem” allows detailed study of how different lineages implement their fitness in a given context.

We bring here examples of model settings allowing, in further research, detailed studies of ontogenies and ecologies on the dish.

## Methods

### Media

**PB :** phosphate buffer as described in Rieger *et al.*[20].**NA:** Nutrient Agar No2 (Imuna Pharm a.s.,) supplemented. For growth in suspensions Nutrient broth No2 (**NB**) was used (Imuna Pharm a.s.,), of identical composition, but without agar.

**NAG**: NA enriched with glucose (Sigma; 0.27 mM; 2.7 mM; 27 mM; 54 mM). In some experiments, NA was enriched with manitol (Sigma; 27 mM), sorbitol (Sigma; 27 Mm), or 6% (w/v) polyethylene glycol (Sigma; mw 6000). In all such cases, the osmotic potential was identical: 0.08 MPa.

Analogically, glucose-enriched broth (**NBG**) was used for cultivations in suspension.

**TN**: 10 g Trypton (Difco), 5 g NaCl (86 mM), 1.5% Agar (Oxoid No 1). Add 1000 ml H_2_O.

**Minimal medium MM:** 21 mM KH_2_ PO_4_, 48 mM Na_2_HPO_4_, 8 mM NaCl, 18 mM NH_4_Cl, 3.9 mM MgSO_4_, 27 mM glucose.

**Minimal medium MMA:** 1.5% agar in MMA.

### Bacteria

The strain *S. rubidea* here labeled **R** was obtained from the collection of the Department of Genetics and Microbiology, Faculty of Sciences, Charles University. The strain *S. marcescens CNCTS 5965* was obtained from the Czech National Institute of Health [20].

The identity of strains was confirmed by MALDI - TOF method, using Bruker Daltonik MALDI Biotyper (performed by A. Nemec, National Health Institute, Prague); the scores assigned to particular strains of *S. rubidaea* (R = 2.241, W = 2.214) and *S. marcescens* (F = 2.151, Fw = 2.212 and M = 2.168) indicate very high probability of correct determination.

It is to be stated that in the previous work, the morphotypes **F** and **Fw** were erroneously determined as belonging to *S. rubidaea* species. In the light of the present, more reliable knowledge, the determination in that paper should be reconsidered – albeit this change has no influence on the results obtained.

The morphotype **M** of *S. marcescens* is a derivative of **F**. It was obtained after many repeated attempts to grow the **F** morphotype in suspensions in the minimal medium MM.

***E. coli*** strain 281 was obtained from the collection of the Department of Genetics and Microbiology, Faculty of Sciences, Charles University.

### Cultivation

If not specified otherwise, bacteria were grown at NAG at 27°C in sealed boxes with controlled humidity. Stabilates were kept at −80°C [20].

New colonies were initiated as follows: (1) as clones from single cells, by classical *sowing* of bacterial suspension (in phosphate buffer); (2) planted by *dropping* dense suspension (10^8^/ml) on a defined place (diameter about 2 mm); (3) planted by *dotting* from material taken by a sterile needle from an older body; (4) by smearing (to grow maculae): 30 μl of bacterial suspension (approx. 10^8^ cells) was applied to a line of approx. 5 cm.

For conditioned agar see [3].

### Documentation

Plates were photographed *in situ* using Olympus C-5050ZOOM digital camera under ambient or penetrating light (Fomei, LP-400 light panel, cold cathode light) or under magnification using a binocular magnifier [3].

Colony margins were observed with fully motorized microscope stand IX81 (Olympus) equipped with objectives LUCPLFLN 20 (NA 0.45) and LUCPLFLN 40 (NA 0.60) and documented with the camera HAMMATSU Orca, with differential interference contrast. Digital images were further elaborated by the software Olympus CELL^R SYSTEM.

Figures shown were selected from an extensive collection of primary photos from several repetitions (5 and more) of each experiment.

Photoshop software was used to assemble the plates as they appear in Figures. No image doctoring was performed except automatic adjustment of brightness and contrast in some cases.

## Competing interests

The authors declare that they have no competing interests.

## Authors’ contributions

IP, JC, and TR contributed equally to the designing and performing the experiments and interpreting their results; AB participated in experiments and data interpretation and provided basic technical support; ZN and AM participated in study design and data interpretation and drafted the paper. All authors have read and approved the final manuscript.
